# Preferential renal tubular injury and increased renal sympathetic activity occur during the early stages of DOCA‐salt hypertension in male rats

**DOI:** 10.14814/phy2.70969

**Published:** 2026-06-10

**Authors:** Edina Da Luz Abreu, Rafael S. Carvalhal, Ana Caroline Marreiros, Stephanie F. Gerolin, Laura Barroso F. Oliveira, Maria Eduarda Abreu‐Cunha, Diogo B. Peruchetti, Cristiane D. Gil, Cássia T. Bergamaschi, Ruy R. Campos

**Affiliations:** ^1^ Cardiovascular Division, Department of Physiology Escola Paulista de Medicina, Universidade Federal de São Paulo São Paulo São Paulo Brazil; ^2^ Renal Physiology Lab, Department of Physiology and Biophysics Institute of Biological Sciences, Federal University of Minas Gerais Belo Horizonte Minas Gerais Brazil; ^3^ Histology and Structural Biology Division, Department of Morphology and Genetics, Escola Paulista de Medicina Universidade Federal de São Paulo São Paulo São Paulo Brazil

**Keywords:** baroreflex, hypertension, kidney injury, urinary biomarkers

## Abstract

Although renal dysfunction is present in arterial hypertension, the mechanisms underlying renal glomerular and tubular alterations remain incompletely understood. We investigated kidney changes during the progression of DOCA‐salt hypertension, on renal sympathetic nerve activity (rSNA), its baroreceptor control, and markers of kidney function. Male Charles River rats were divided into independent experimental groups: controls (UNx), hypertensive (DOCA) rats treated for 3 and 5 weeks. All the animals underwent nephrectomy. Mean arterial pressure (MAP) and heart rate (HR) in awake rats, and rSNA and its baroreflex in anesthetized rats. Urinary biomarkers, total protein, gamma‐glutamyl transpeptidase (GGT), lactate dehydrogenase (LDH), epidermal growth factor (EGF), cystatin C, β2‐microglobulin (β2M), alpha‐1 acid glycoprotein (AGP), neutrophil gelatinase‐associated lipocalin (NGAL), and albumin and renal morphology were evaluated. Baseline rSNA was elevated after 3 weeks of treatment and remained increased at 5 weeks, associated with an increase in MAP and reduced baroreflex sensitivity. Urinary LDH and GGT markers were higher in hypertensives, with GGT remaining elevated. AGP, NGAL, and albumin levels were unchanged. EGF, β2M, and particularly cystatin C were significantly elevated in 3 and 5 weeks. These findings indicate that preferential renal tubular injury associated with increased rSNA occurs during the early stage of DOCA‐salt hypertension.

## Graphical abstract

Evaluation of cardiovascular parameters and renal mediators during the development of arterial hypertension in male DOCA‐Salt rats.

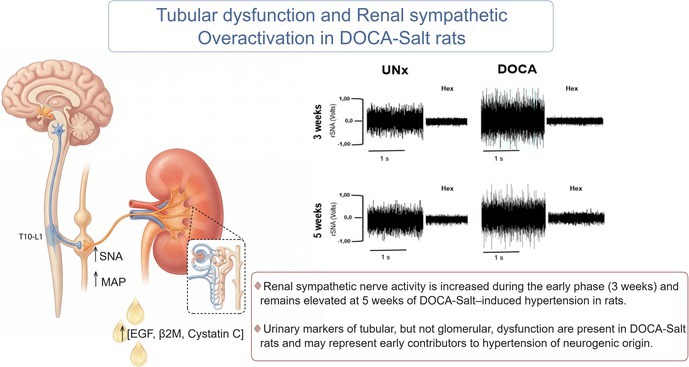

## 1 INTRODUCTION

In recent years, it has become evident that kidneys play a role that goes beyond regulating body fluids. They act directly in signaling between the peripheral and central nervous systems, establishing a bidirectional relationship that results in a progressive vicious cycle in pathophysiological states (Baumann et al., 2024; Jones et al., 2025; Lincevicius et al., 2017; Sales da Silva et al., 2020). However, the underlying mechanisms that combine kidney damage, sympathetic overactivation to the kidney, and hypertension remain uncertain, as does when glomerular and tubular lesions begin.

The bidirectional effect of persistently high blood pressure can accelerate the progression of kidney dysfunction, and the progressive decline in glomerular filtration rate can, conversely, interfere with achieving adequate blood pressure control. Thus, the coexistence of uncontrolled hypertension and amplified kidney damage increases the risk of cardiovascular disease and subsequent development of various organ lesions (Weldegiorgis & Woodward, 2020). In this way, establishing early renal biomarkers associated with the onset of cardiovascular diseases and their contribution is necessary. However, it has not yet been possible to find a specific biomarker, especially for the resistant and refractory form of hypertension. The sympathetic nervous system plays a central role in the development and maintenance of hypertension, in part through increased renal sympathetic nerve activity (rSNA). Antihypertensive therapies targeting sympathetic modulation are often insufficient to adequately suppress sympathetic outflow. In this context, renal nerve denervation has emerged as a therapeutic alternative or adjunct strategy to treat resistant hypertension (Cluett et al., 2024).

Several studies show that increased sympathetic nerve activity (SNA) is a possible cause of hypertension, through mechanisms that are still unknown. Furthermore, increased dietary sodium intake affects both blood pressure and SNA (Banek et al., 2016; Baumann et al., 2024; Lauar et al., 2024). The sympathetic imbalance may arise from adverse renal events that affect the function of brain areas involved in blood pressure and SNA control (Banek et al., 2019; Campos et al., 2015; Sealey & Blumenfeld, 2025). In fact, it has recently been demonstrated that total renal denervation or only the removal of renal afferents by inactivating TRPV1 receptors reduced blood pressure in a DOCA‐salt hypertension model in rats (Banek et al., 2016; Lauar et al., 2024). However, there is currently no information about the renal mechanisms involved in this phenomenon, including the control of tubular and glomerular functions. Part of the present study was designed to address this topic.

Tubular atrophy and interstitial fibrosis impair key mechanisms underlying blood pressure regulation, including solute transport and hormonal control of circulating volume. These changes are associated with reduced renal function and hypertension (Schnaper, 2017). Nevertheless, biomarkers reflecting tubular injury and glomerular dysfunction throughout the progression of DOCA‐salt hypertension remain insufficiently characterized. Although not yet widely adopted in clinical practice, urinary biomarkers such as cystatin C, β2‐microglobulin (β2M), and other low‐molecular‐weight proteins, including epidermal growth factor (EGF), are considered indicators of glomerular filtration and tubular reabsorption (Bonnard et al., 2023; Brobak et al., 2024; Pavlov et al., 2013).

The DOCA‐salt hypertension model in rats incorporates features observed in the etiology of human hypertension, including elevated rSNA and salt sensitivity (Brobak et al., 2024). Thus, it constitutes an interesting model for investigating the direct relationship between an increase in rSNA, renal dysfunction, and hypertension. Thus, this study was designed to explore potential modifications in renal biomarkers associated with glomerular and tubulointerstitial injury during the development of DOCA‐salt hypertension. Considering the importance of sympathetic overactivation in this model, direct rSNA was quantified, as well as its control by arterial baroreceptors in both early and later stages of hypertension development in DOCA‐salt rats.

## 2 MATERIALS AND METHODS

Male *Wistar* Charles River rats, 7 weeks old (180–220 g), were housed in group cages in accordance with a protocol approved by the Ethics Committee on Animal Use (CEUA) of the Universidade Federal de São Paulo (CEUA/Unifesp, process no. 3812260824). All experimental approaches applied in this study followed the guidelines recommended by the National Council for the Control of Animal Experimentation (CONCEA, Brazil) and the National Institutes of Health (NIH, 1996). The animals had free access to food and water ad libitum and were kept in a temperature‐controlled environment (21°C ± 1°C, 65% humidity).

All chemicals used in the present study were of analytical grade. Ketamine hydrochloride (Syntec®, SP, Brazil), xylazine (Sespo® Indústria e Comércio Ltda, SP, Brazil), urethane, and deoxycorticosterone acetate (DOCA) were obtained from Sigma‐Aldrich Co®, USA, codes: U2500 and D7000, respectively.

### 2.1 Experimental protocols

The experiments were performed on male rats, divided into UNx (treated with vehicle for 3 and 5 weeks) and DOCA (treated with DOCA‐salt for 3 and 5 weeks), 7 days after surgical recovery. All animals, including the controls (UNx), underwent right unilateral nephrectomy.

Two independent series of experiments were performed. In the first series of experiments, the rats were placed in individual metabolic cages for 24 h as an adaptation period (Nalgene, Tecniplast®, Buguggiate, Italy). After 24 h, urine was collected and clarified, then centrifuged 5 times at 10.000 rpm for 20 min and stored at −80°C for biochemical and multiplex analyses. Next, the rats were anesthetized under ketamine and xylazine (100 and 10 mg/kg, i.p.) anesthesia, and the kidneys were surgically excised for histomorphometric analysis. In the second experimental series, the rats were anesthetized with urethane (1.4 g/kg, i.v.), and the cardiovascular parameters, mean arterial pressure (MAP), heart rate (HR), renal sympathetic nerve activity, and renal sympathetic baroreflex sensitivity were assessed.

The DOCA Salt rat model was developed as per previous reports (Basting & Lazartigues, 2017; Pestana‐Oliveira et al., 2020; Selye & Stone, 1946; Tostes Passaglia et al., 2000). Initially, the animals from all groups underwent surgery for uninephrectomy. Rats were previously relaxed with acepromazine (0.2 mg/kg i.p.) (Dechra® Ltda, Brazil) and then briefly anesthetized with ketamine (80 mg/kg, i.p.) and xylazine (3 mg/kg i.p.) to undergo removal of the right kidney. They receive analgesia with ketoprofen (0.2 mg/kg s.c.) for 72 h following the procedure. After 7 days of post‐surgery, the rats were randomized into UNx (Control) and DOCA‐salt (Hypertensive) groups. All the animals were fed commercial rat food (Nuvilab®, Quintia S/A, Colombo, Brazil). From then on, the DOCA animals receive weekly subcutaneous injections of deoxycorticosterone acetate, diluted in mineral oil and propylene glycol (1:1) at doses of 20 mg/kg in the 1st week, 12 mg/kg in the 2nd and 3rd weeks, and 6 mg/kg from the 4th week until the end of the experimental period (Abreu et al., 2022; Tomazelli et al., 2023; Wenceslau & Rossoni, 2014). Additionally, the DOCA rats received daily water supplemented with sodium chloride (1%) + potassium chloride (0.2%). The control group receives only the dilution vehicle solution and the drinking water. The control group was followed for 3 or 5 weeks, matched to the DOCA‐salt group at the same time points (3 and 5 weeks).

### 2.2 Mean arterial pressure (MAP), heart rate (HR), renal sympathetic nerve activity (rSNA) recordings, and the percentage of the maximal sympathetic response

The femoral vein and artery were catheterized under ketamine and xylazine (100 and 10 mg/kg, i.p.) anesthesia. Twenty‐four hours later, the MAP and HR were assessed in conscious rats. Then, the basal levels of rSNA (spikes/s), its baroreflex sensitivity, and the maximum rSNA response were measured in urethane‐anesthetized rats (1.4 g/kg, i.v.). Animals were mechanically ventilated with a tidal volume of 1 mL/100 g of body weight and a respiratory rate of approximately 80 breaths/min. The value of basal renal sympathetic nerve activity relative to its maximum response was used to normalize the data and allow comparison of rSNA levels between individuals (Sharma et al., 2017). To determine the maximum rSNA response, acute hypoxia (60 s) was induced at the end of the experiments. The basal rSNA value was calculated for each rat relative to its maximum response (basal rSNA × 100/maximum rSNA) and expressed as a percentage of the maximum response. At the end of the experiments, the animals were already deeply anesthetized, and an overdose of urethane was administered intravenously.

### 2.3 Baroreflex control of renal sympathetic nerve activity (rSNA)

Rats were slowly anesthetized with urethane (1.4 g/kg, i.v.). A tracheotomy was performed to reduce airway resistance. Body temperature was maintained at 37°C using a servo‐controlled heating blanket (Letica Scientific Instruments®, USA). The flank incision was performed to expose the left renal artery via a retroperitoneal approach. In the same incision, a section of the left renal nerve was isolated on the renal artery and placed on the bipolar silver electrodes. Baroreceptor control of rSNA was evaluated via intravenous infusion of vasoactive drugs. Phenylephrine hydrochloride (Sigma‐Aldrich Co®, USA, codes: P6126) (10 μg/kg/2.0 mL/min) and Sodium nitroprusside (Sigma‐Aldrich Co®, USA, codes: PHR1423) (20 μg/kg/0.5 mL/min) were infused to increase or decrease arterial pressure, respectively. Values of matching MAP variations (±40 mmHg) with reflex rSNA response were plotted separately for each vasoactive drug at every 5 mmHg of MAP change to create linear regression curves of the baroreceptor function for each group, and their slopes (spikes/s/mmHg) were compared to evaluate changes in baroreflex sensitivity among groups.

#### 2.3.1 Assessment of biomarkers of kidney function

Pre‐clarified 24‐h urine samples were used to measure activity of lactate dehydrogenase (LDH, a marker of kidney cell injury) and gamma‐glutamyl transferase (GGT, a marker of proximal tubule epithelial cell injury); total protein, creatinine, and urea were acquired from LabTest® (references: n° 86, n° 105, n° 99, n° 35, n° 27, respectively). Enzyme activity (LDH and GGT) data are expressed in U/L. Total protein, creatinine, and urea are expressed in mg/dL. All data are normalized to the 24‐h urine volume.

### 2.4 MILLIPLEX® tests of kidney function by urinary markers

The levels of EGF, Cystatin C, ß2M, AGP, Lipocalin‐2/NGAL, and Albumin urinary were measured by the method fluorometric Luminex® with the commercial MILLIPEX (Merck Millipore®, codes: RKTX2MAG‐37K, kit Rat Kidney Toxicity Panel 2). Data are expressed in ng/mL corrected for 24‐h urine volume.

### 2.5 Histomorphometric analysis

Renal tissue samples were fixed in 4% paraformaldehyde for 48 h and embedded in paraffin. Kidney sections (5 μm thick) were stained with periodic acid‐Schiff (PAS) as described above (Moraes et al., 2025; Peruchetti et al., 2021; Teixeira et al., 2020) for investigating structural changes. Fifteen images in total for each biological sample were randomly acquired using a Zeiss® AXIO SCOPE.A1 microscope (Carl Zeiss, Jena, Germany) with ×20 or ×40 objective from different cortical and medullary regions of the kidney, followed by quantification and analysis using Image‐Pro Plus software (National Institutes of Health, Bethesda, MD, USA). To evaluate possible changes in the glomerular structure, it was assessed: (1) glomerular cellularity (number of cell nuclei at glomerular tuft) (12–15/group); (2) mesangial expansion [PAS‐stained area (strong purple color area) normalized by glomerular tuft area] (12–15/group); (3) Bowman's capsule space area (white space area normalized by total glomerular area) (12–15/group); (4) glomerular tuft area (expressed as percentage of the total glomerular area) (12–15/group) and (5) the total glomerular area (expressed as pixels) (*N* = 12–15/group). The tubules were evaluated according to: (1) tubular epithelial cell height (measuring the straight‐line distance from brush‐border (BB) apical membrane up to tubular basal membrane) (12–15/group); (2) number of interstitial cells (number of interstitial cell nuclei normalized by total cell nuclei) (12–15/group); (3) total tubular cells (number of BB‐positive cell nuclei by field) (12–15/group) and (4) tubular (intra‐tubular) area (tubular lumen area normalized by respective tubule area) (12–15/group). All analyses were conducted blindly.

### 2.6 Statistical analysis

GraphPad Prism 8® software was used for statistical analysis, and the normality test was assessed using the Shapiro–Wilk test. All pooled data presented in the figures were expressed as mean ± standard deviation (SD). Differences between groups were analyzed using two‐way analysis of variance (ANOVA). When differences were indicated, the Bonferroni post hoc test was employed. Statistical significance was set at *p* < 0.05.

## 3 RESULTS

### 3.1 Baseline values of MAP, HR, rSNA (basal and maximal induced response), in DOCA‐salt hypertension

**FIGURE 1:**
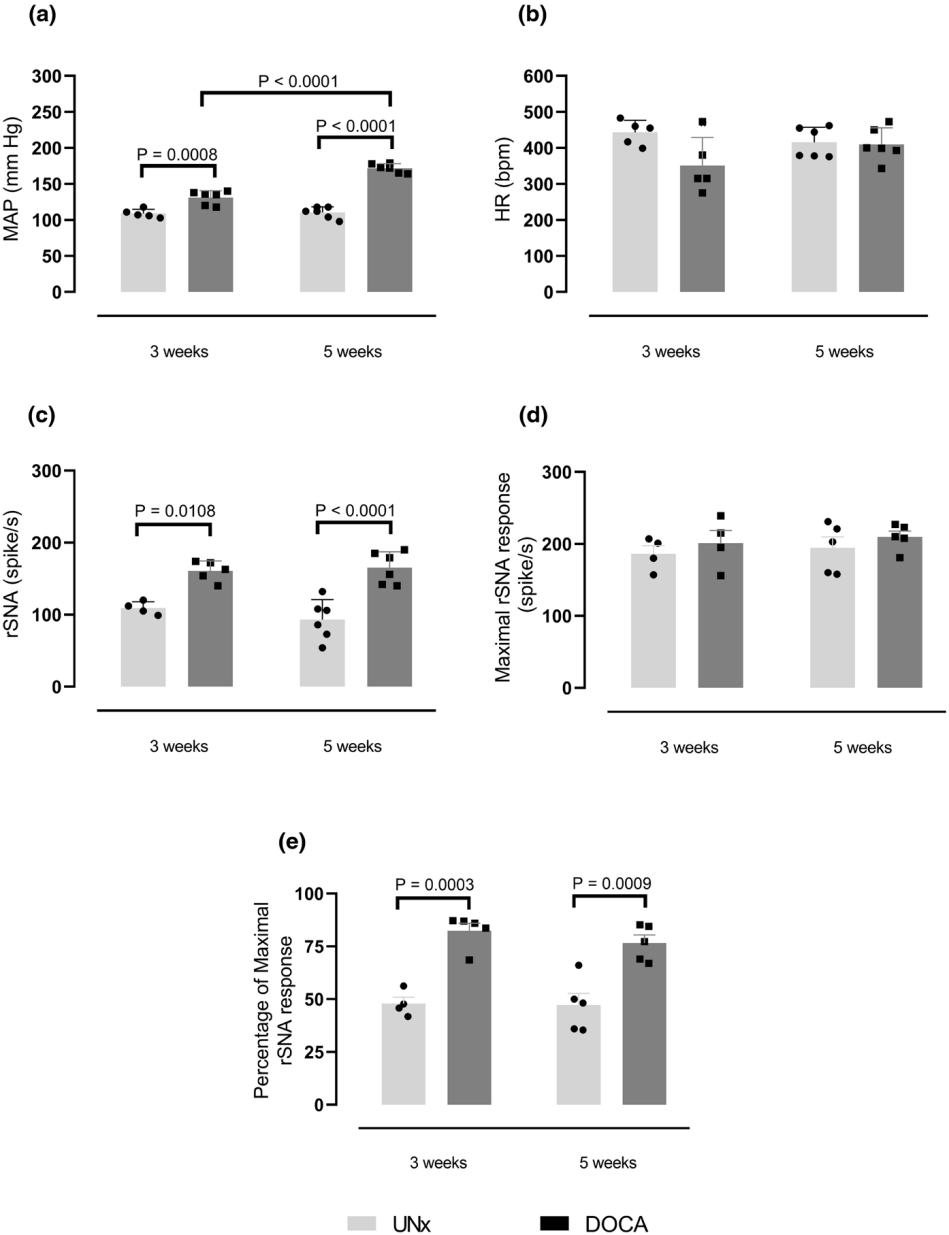
Cardiovascular and autonomic function in DOCA‐Salt rats after 3 and 5 weeks of DOCA‐Salt treatment. Values are presented as mean ± SD; *p* < 0.05 was considered statistically significant. Two‐way ANOVA with Bonferroni post hoc test. UNx, control group; DOCA, hypertensive group. (a) MAP, mean arterial pressure; (b) HR, heart rate; (c) rSNA, renal sympathetic nerve activity; (d) maximum response of rSNA (spikes/s); and (e) percentage of rSNA maximum response based on baseline values. (*N* = 5‐6/group).

MAP of awake animals was significantly increased in the DOCA groups compared with UNx rats at both time points (3 weeks: 110 ± 5.7 to 134* ± 7.9, *p* = 0.0008; 5 weeks: 106 ± 5.5 to 169*^#^ ± 9.6 mmHg, *p* = <0.0001, Figure 1a, *n* = 6). In addition, MAP was significantly higher in the DOCA group at 5 weeks than at 3 weeks of hypertension induction (Figure 1a, *p* = < 0.0001). Baseline HR did not differ among groups or experimental periods (3 weeks: 439 ± 32.7 to 345 ± 76.0, *p* = 0.0795, *n* = 5; 5 weeks: 406 ± 39.40 to 418 ± 43.9 bpm, *p* = >0.9999, Figure 1b, *n* = 6). DOCA‐salt treatment induced a significant increase in resting rSNA compared with the control group at both 3 weeks (110 ± 8.7 to 158* ± 12.5, *p* = 0.0108, Figure 1c, *n* = 5) and 5 weeks (80 ± 19.0 to 165* ± 9.6 spike/s, *p* = <0.0001, Figure 1c, *n* = 6). However, the maximum absolute values of rSNA responses induced by acute hypoxia did not differ among groups (Figure 1d, *n* = 4–5). Yet, when baseline rSNA values are expressed as a percentage of the maximum response, a statistically significant increase is observed from the third week of treatment compared to UNx rats (*p* = 0.0003), with no difference detected between the third and fifth weeks of treatment, indicating that rSNA is already elevated in the early stages of hypertension development (Figure 1e, *n* = 5).

### 3.2 Baroreflex control of renal sympathetic nerve activity (rSNA)

**FIGURE 2:**
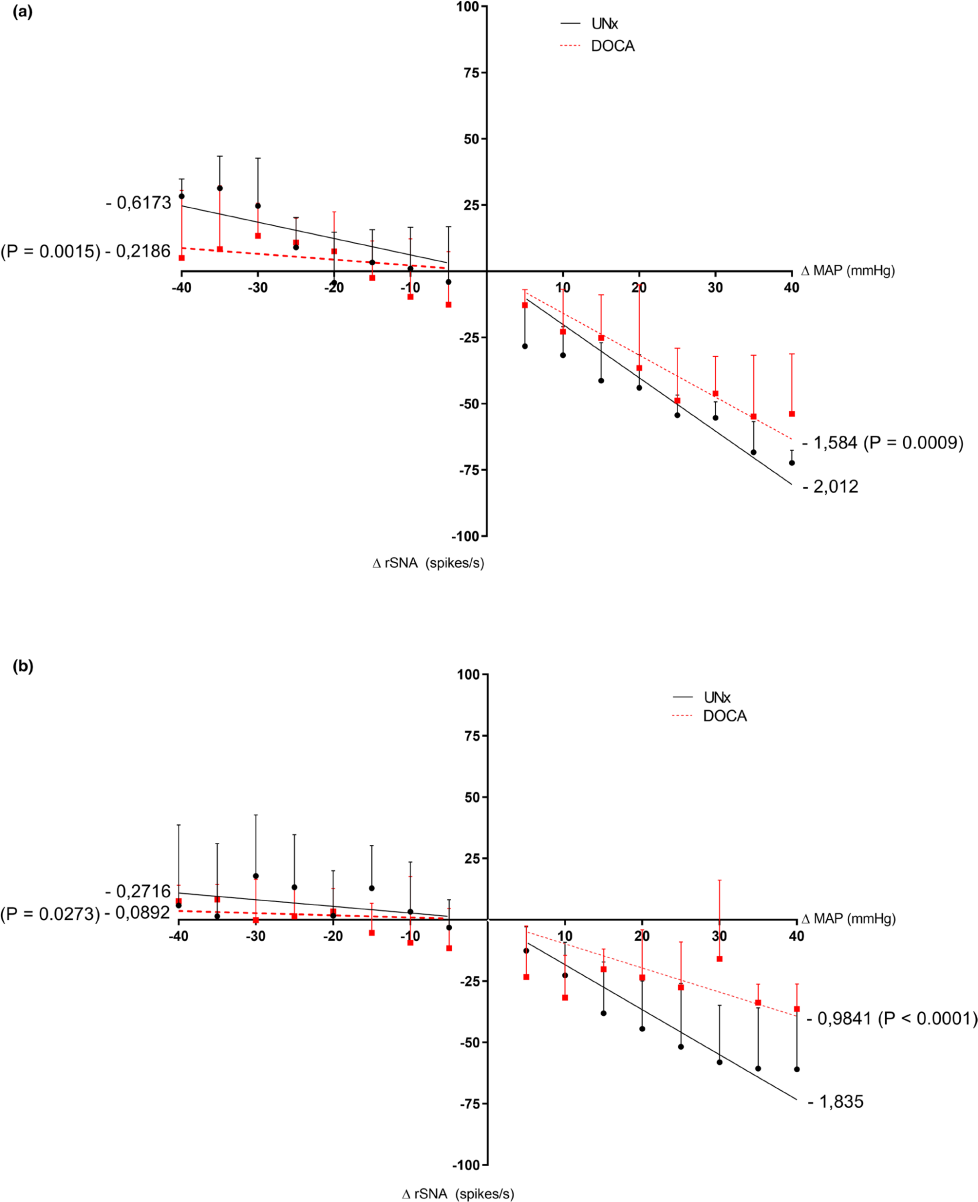
Baroreflex Sensitivity of Renal Sympathetic Nerve Activity in DOCA‐Salt rats. (a) 3 weeks of induction; (b) 5 weeks of induction. Upper panel, curve of baroreflex function, evaluated by variation of rSNA for every 5 mmHg of MAP reduction induced by continuous infusion of sodium nitroprusside; Bottom panel, curve of baroreflex function, evaluated by variation of rSNA for every 5 mmHg of MAP increase induced by continuous infusion of phenylephrine. rSNA, renal sympathetic nerve activity; MAP, mean arterial pressure; spikes/s, spikes per second. Values are presented as mean ± SD; *p* < 0.05 was considered statistically significant. Two‐way ANOVA with Bonferroni post hoc test. UNx, control; DOCA, hypertensive. (*N* = 3–6/group).

Baroreflex control of rSNA was already impaired after 3 weeks of DOCA treatment and remained compromised at 5 weeks, as shown in Figure 2a (right panel). DOCA‐treated rats at 3 weeks exhibited impaired arterial baroreflex control of rSNA in response to phenylephrine‐induced pressor responses compared with UNx controls. This impairment is reflected in a reduced slope of the baroreflex sensitivity curve (UNx: −2.012 ± 0.12, *n* = 3 vs. DOCA: −1.584 ± 0.11 spikes/s/mmHg, *n* = 6, *p* = 0.0009). Similarly, the depressor response induced by sodium nitroprusside was attenuated in the same animals (Figure 2a, left panel), as indicated by a reduced slope for each 5 mmHg change in MAP (UNx: −0.617 ± 0.12, *n* = 3 vs. DOCA: −0.219 ± 0.10 spikes/s/mmHg, *n* = 6, *p* = 0.0015), indicating that the control of rSNA by the baroreceptors is already impaired in the early stages of hypertension development.

Figure 2b shows that baroreflex impairment persisted in DOCA‐treated rats after 5 weeks of treatment. The pressor response induced by phenylephrine remained significantly blunted (UNx: −1.835 ± 0.11, *n* = 5 vs. DOCA: −0.941 ± 0.13, spikes/s/mmHg, *n* = 5; Figure 2b, *p* < 0.0001, right panel). Likewise, the depressor response to sodium nitroprusside was nearly abolished (UNx: −0.272 ± 0.14, *n* = 5 vs. DOCA: −0.008 ± 0.10 spikes/s/mmHg, *n* = 6; Figure 2b, *p* = 0.0273, left panel). Comparison of the slopes in the control group shown in Figure 2a,b revealed no statistically significant differences (data not shown).

### 3.3 Biochemical markers of kidney function

**FIGURE 3:**
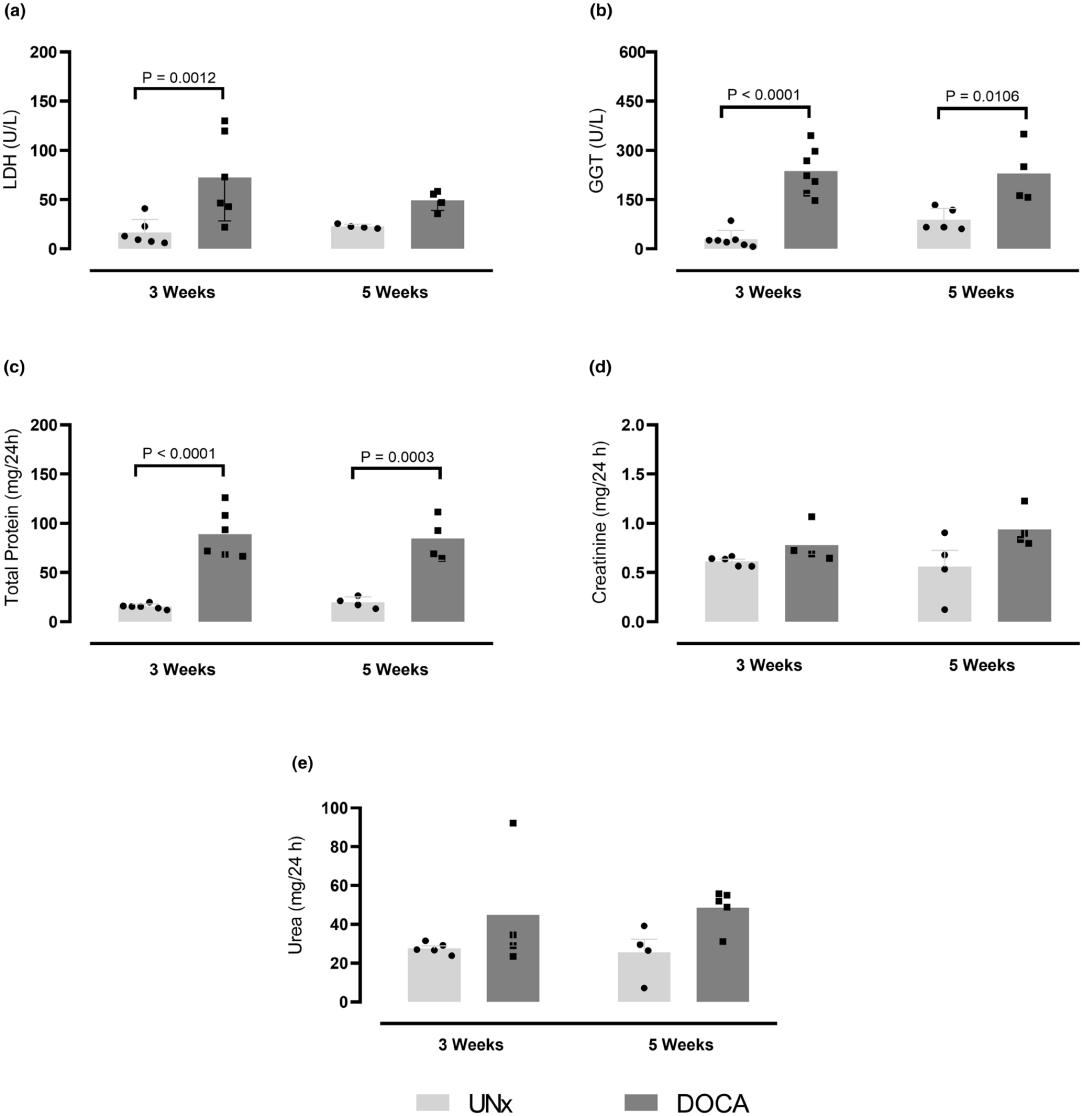
LDH and GGT activity, total protein, creatinine, and urea urinary levels in DOCA‐salt‐induced hypertensive rats at 3 and 5 weeks. (a) LDH, (b) GGT, (c) total protein, (d) creatinine, and (e) urea. Values are presented as mean ± SD; *p* < 0.05 was considered statistically significant. Two‐way ANOVA with Bonferroni post hoc test. UNx, control; DOCA, hypertensive. LDH, lactate dehydrogenase; GGT, gamma‐glutamyl transferase. (*N* = 4–6/group).

The activities of LDH and GGT were increased by 3 weeks of treatment in DOCA‐salt rats to 72.30 ± 40.11, *p* = 0.0012, *n* = 6, and 236.70 ± 65.50 U/L, *p* = <0.0001, *n* = 7, respectively, from 16.6. ± 12.22 and 29.40 ± 24.22 U/L, *n* = 5, in the corresponding control (Figure 5a,b). After 5 weeks of treatment, even with a slight reduction, GGT activity remained elevated in DOCA‐salt rats (229.7 ± 78.49 U/L, *p* = 0.0106, *n* = 5), compared to the UNx group (88.8 ± 30.70 U/L, *n* = 4) (Figure 3a,b). While LDH showed a reduction in 5 weeks compared to the 3‐week animals, it was still higher than its corresponding control (Figure 3a,b). In addition to this data, an increase in urinary total protein levels was also observed in hypertensive animals in both experimental periods when compared to control animals (3 weeks: 15.50 ± 2.45 to 89.0 ± 22.29, *p* = <0.0001, *n* = 6; 5 weeks: 19.60 ± 4.96 to 84.40 ± 18.98, *p* = 0.0003, *n* = 4) (Figure 3c). However, there was no difference in urinary creatinine and urea levels among the groups at any of the time points evaluated in this study (Figure 3d,e).

### 3.4 Multiplex assay of renal function biomarkers

**FIGURE 4:**
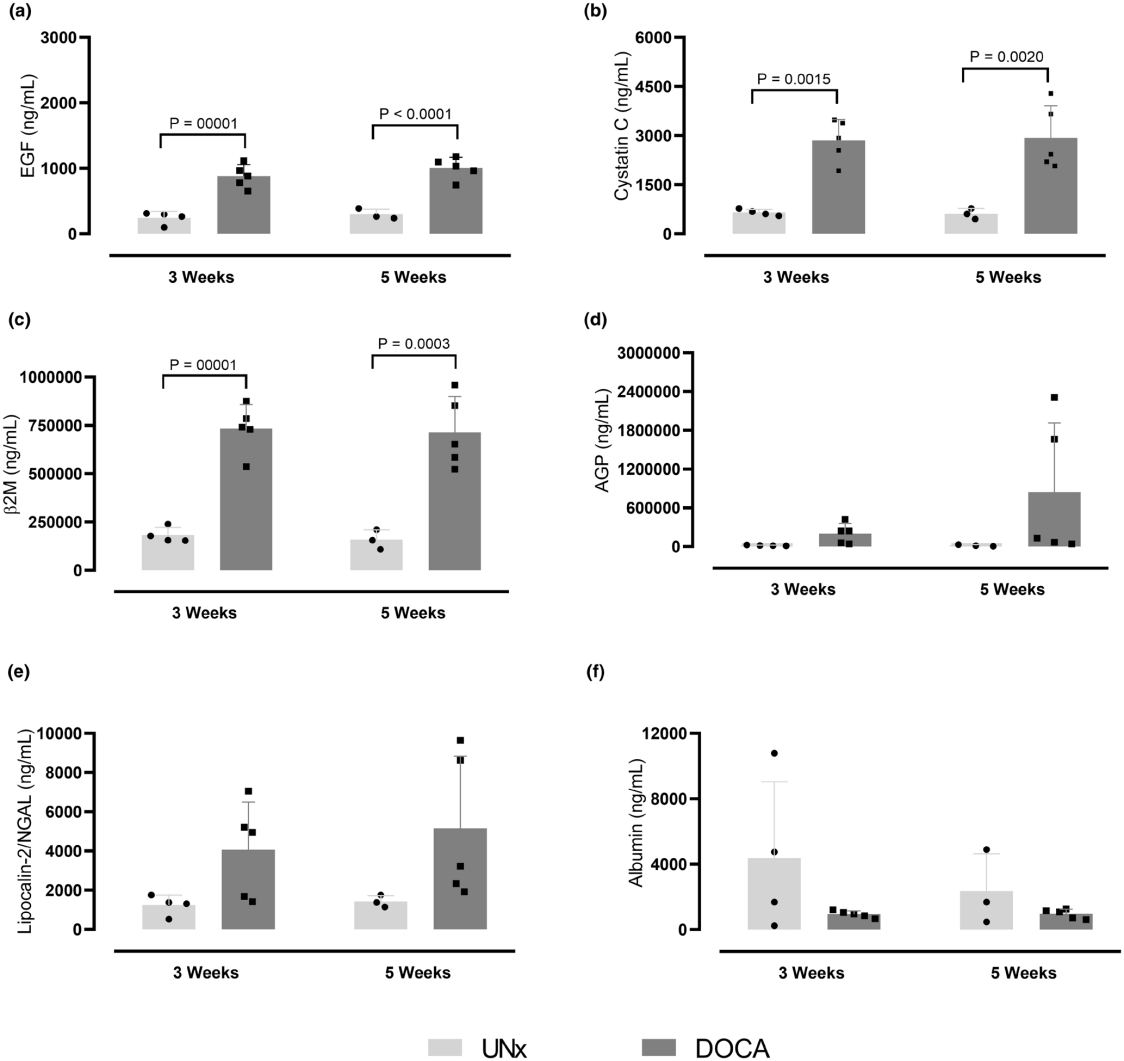
Levels of EGF, Cystatin C, ß2M, AGP, Lipocalin‐2/NGAL and Albumin urinary in DOCA‐salt hypertensive rats at 3 and 5 weeks of treatment. (a) EGF, (b) Cystatin C, (c) ß2M, (d) AGP, (e) Lipocalin‐2/NGAL and (f) Albumin. Values are presented as mean ± SD; *p* < 0.05 was considered statistically significant. Two‐way ANOVA with Bonferroni post hoc test. UNx, control; DOCA, hypertensive. EGF, epidermal growth factor; Cystatin C, cystatin C; ß2M, beta 2 microglobulin; AGP, alpha‐1 acid glycoprotein; Lipocalin‐2/NGAL, neutrophil gelatinase‐associated lipocalin 2 and Albumin, albumin. (*N* = 4–6/group).

DOCA‐salt animals showed high levels of EGF, Cystatin C, and ß2M (Figure 4a–c) after 3 weeks and 5 weeks of treatment (3 weeks: 880.2 ± 156.6, *p* = 0.0001; 2853.0 ± 591.40, *p* = 0.0015; 733805.1 ± 111160.3, *p* = 0.0001 ng/mL, *n* = 5 vs. UNx: 243.3 ± 85.40; 653.6 ± 83.40; 182219.4 ± 34231.60, *n* = 4 plus 5 weeks: 1003.7 ± 147.3, *p* = < 0.0001; 2929.8 ± 882.10, *p* = 0.0020; 714781.3 ± 164932.60, *p* = 0.0003, ng/mL n = 5 vs. UNx: 297.9 ± 64.00; 612.6 ± 132.00; 158648.6 ± 41621.20 ng/mL, *n* = 3). However, the levels of AGP and Albumin were not different among groups (Figure 4d,f). Although Lipocalin‐2/NGAL levels did not show a significant statistical difference, they were higher in animals treated with DOCA, both after 3 and after 5 weeks of treatment (Figure 4e).

### 3.5 Histomorphometry analysis

**FIGURE 5:**
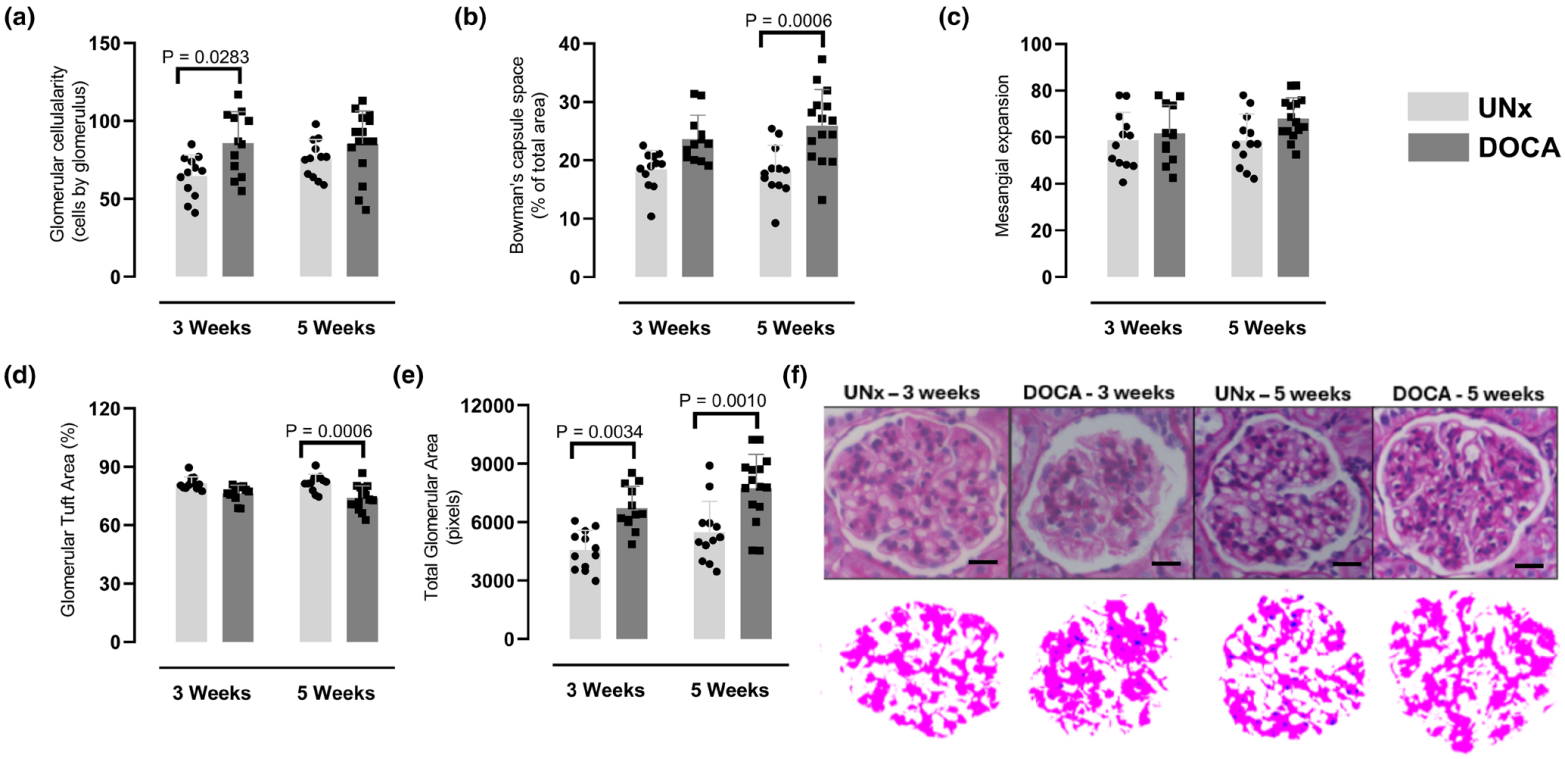
Glomerular and mesangial analysis in longitudinal sections stained with periodic acid‐Schiff (PAS) to glomerular cellularity, Bowman's capsule space area, mesangial expansion, percentage of tuft area, and total glomerular area. Scale bars, 50 μm. (a) Glomerular cellularity (b), Bowman's capsule space (c), mesangial expansion, (d) percentage glomerular tuft area (e) total glomerular area, and (f) representative images. Values are presented as mean ± SD; *p* < 0.05 was considered statistically significant. Two‐way ANOVA with Bonferroni post hoc test. UNx, control; DOCA, hypertensive. (*N* = 12–15/group).

**FIGURE 6:**
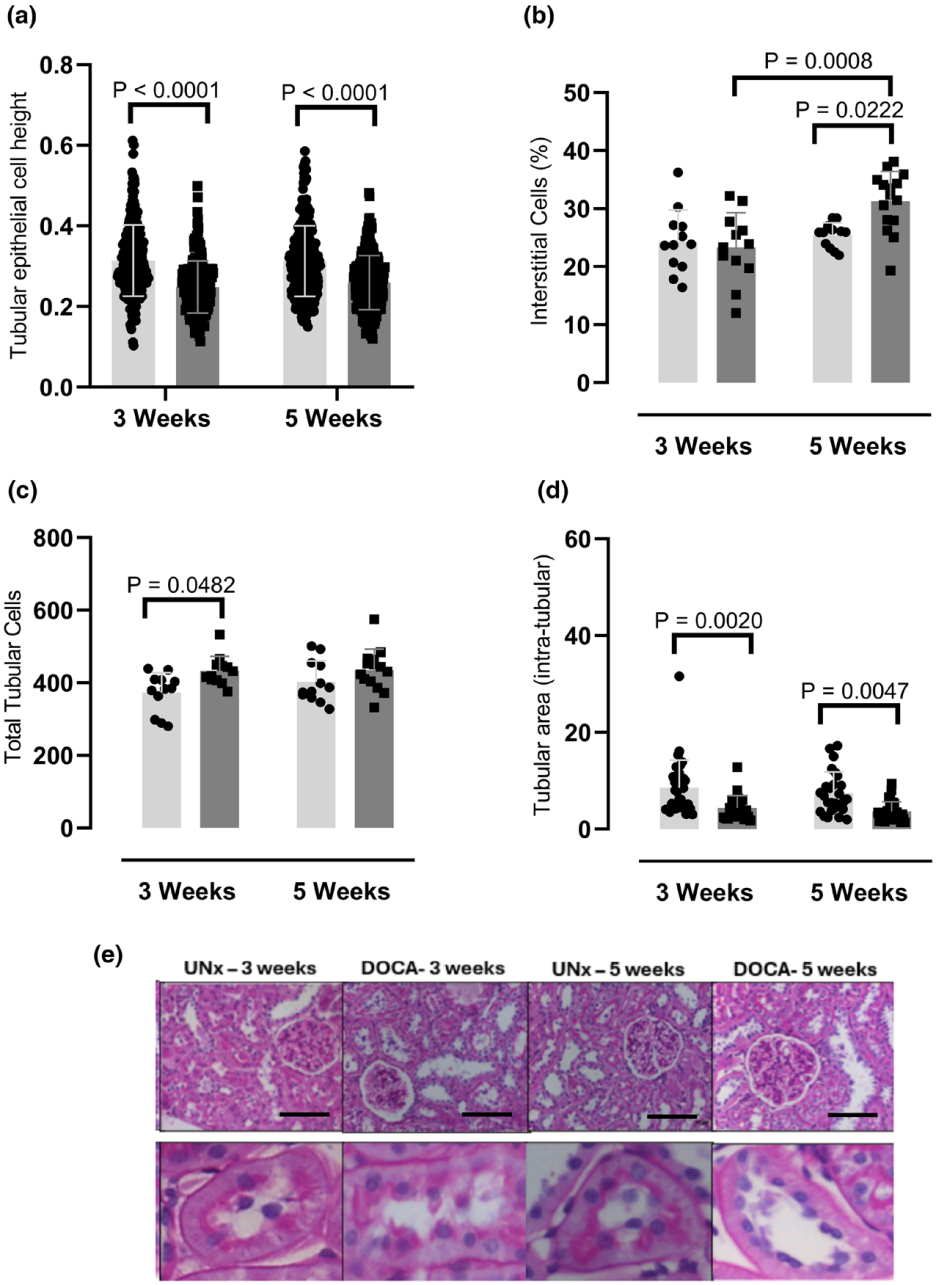
Evaluation of changes in tubules in longitudinal sections stained with periodic acid‐Schiff (PAS) to tubular epithelial cell height, number of interstitial cells, total tubular cells, and tubular (intra‐tubular) area. Scale bars, 100 μm. (a) tubular epithelial cell height, (b) interstitial cells, (c) total tubular cells, (d) tubular (intra‐tubular) area, and (e) representative images. Values are presented as mean ± SD; *p* < 0.05 was considered statistically significant. Two‐way ANOVA with Bonferroni post hoc test. UNx, control; DOCA, hypertensive; NC, negative control (kidney collected on the day of nephrectomy). (*N* = 12–15/group).

Changes in glomerular morphometric parameters, such as Bowman's capsule space and total glomerular area, were observed in the DOCA group compared to the UNx group after 3 and 5 weeks of treatment (Figure 5b,e, *n* = 12–15). Furthermore, when compared to UNx animals treated for 5 weeks, DOCA‐salt rats showed a greater percentage of Bowman's capsule space and total glomerular area with a significant reduction in tuft size (Figure 5b,d,e). Animals treated with DOCA for 3 weeks showed a higher number of glomerular cells (Figure 5a). However, no changes in mesangial expansion were observed in the groups and time points investigated (Figure 5c). On the other hand, a reduction in tubular epithelial cell height was observed in DOCA animals at 3 and 5 weeks compared to UNx (Figure 6a). In addition, an increase in the number of interstitial cells was observed in DOCA animals at 5 weeks compared to UNx (Figure 6b). A change in the total number of tubular cells was observed in animals treated with DOCA for 3 weeks (Figure 6c), not differing from those at 5 weeks. However, when the intraluminal area was assessed, DOCA‐salt rats showed a greater reduction compared to the UNx group (Figure 6d).

## 4 DISCUSSION

The development and progression of target organ damage of hypertension have been associated with increased sympathetic activity, particularly in the renal territory. In this study, we demonstrate that during the initial phase of DOCA‐salt‐induced hypertension, renal basal sympathetic activity is increased, accompanied by impaired arterial baroreflex control of this activity. These autonomic changes occur early in the progression of malignant hypertension and provide new evidence of renal injury. The main findings of this study indicate a preferential tubular kidney dysfunction in the early phase of DOCA‐salt hypertension, characterized by elevated LDH and GGT activity and increased total protein levels in the urine after 3 weeks of DOCA‐salt treatment. This is accompanied by an increase in EGF, Cystatin C, and β2M induced by DOCA‐salt treatment, along with structural changes indicative of tubulointerstitial injury. These findings indicate that renal tubular changes associated with increased rSNA in the kidneys occur in stage 1 hypertension (3 weeks) and represent early contributors to neurogenic hypertension.

In fact, increased sympathetic vasomotor activity is a characteristic feature observed in both humans and rodent models of hypertension, and its attenuation leads to substantial improvement of cardiovascular and renal function in hypertensive individuals (Grassi et al., 2025; Nishi et al., 2015). Elevated sympathetic activity in DOCA‐salt animals has been described in other studies (Basting et al., 2018; Guimaraes et al., 2012; Kandlikar & Fink, 2011), but none have evaluated rSNA by direct renal nerve recording at different times of development of DOCA‐salt hypertension. We observed that rSNA increased in stage 1 hypertension. Interestingly, upon reaching stage 2 hypertension, the animals did not show a further increase in rSNA. We can infer that sympathetic hyperactivity results in the worsening of blood pressure levels, just as other factors contribute to the malignant elevation of blood pressure. Associated with rSNA, DOCA animals showed reduced baroreflex sensitivity. In agreement, similar results in baroreflex were also obtained in DOCA‐salt and other models of hypertension (Basting et al., 2018; Carvalho‐Galvão et al., 2018; Guimaraes et al., 2012; Lincevicius et al., 2017; Osis et al., 2021; Zager et al., 2013). Nakamura and colleagues showed that after 7 days of DOCA treatment, a central impairment of arterial baroreceptor reflex control of the splanchnic nerve was recorded between the cardiac and celiac ganglion in urethane anesthetized rats (Nakamura et al., 1988). In the present study, we described an impairment of baroreceptor reflex control of rSNA after 3 weeks of DOCA treatment, indicating that the reflex does not play any inhibitory role in the development of DOCA‐salt hypertension and sympathetic overactivation to the kidneys. In addition to baroreceptor impairment, it has been described that DOCA amplifies the role of NaCl, leading to an increase in sympathetic vasomotor activity (Beckmann et al., 2025; O'Donaughy & Brooks, 2006).

Salt sensitivity, a key element for predisposition and development of many cases of hypertension (Beckmann et al., 2025), is present to a similar extent as that which occurs in human salt‐sensitive hypertension (Basting & Lazartigues, 2017; Pestana‐Oliveira et al., 2020). This increased sensitivity to salt may originate from defective renal mechanisms for handling sodium. Rats subjected to DOCA‐Salt induction and disruption of sympathetic connection via renal denervation showed a selective and sustained reduction in salt appetite and blood pressure (Lauar et al., 2024). Together, these studies highlight the importance of renal sympathetic overactivity (afferent and efferent pathways) in promoting sodium retention and kidney dysfunction.

Furthermore, various stimuli, including hormones and growth factors, regulate epithelial sodium channel (ENaC), thereby fine‐tuning Na^+^ reabsorption in the kidney. Members of the EGF family play important roles in maintaining transepithelial Na^+^ transport; however, whether EGF modulates ENaC—potentially contributing to hypertension—remains unclear.

In addition to these findings, renal tubular epithelial cell damage is a central mechanism in the pathophysiology of tubulointerstitial fibrosis, strongly correlated with declining renal function and hypertension (Nielsen et al., 2017; Osborn & Fink, 2010; Tsuji et al., 2025). EGF levels are related to its receptor, EGFR, which in turn varies with cellular components, tissue type, and pathological condition. In the kidney, EGF/EGFR is expressed in the glomerulus and the tubulointerstitial compartment (Postalcioglu et al., 2024). EGFR may have a dual role in kidney function, acting beneficially under physiological conditions; however, its dysregulation may also be intrinsically involved in mediating cardiovascular and renal pathologies. Indeed, selective deletion of proximal tubule EGFR delayed kidney functional recovery from ischemic injury (Chen et al., 2012), but persistent EGFR activation resulted in the development of tubulointerstitial injury (Tang et al., 2013). In the present study, we did not specifically evaluate the expression of EGFR and its different isoforms, but we observed exacerbated levels of EGF in the urine of DOCA‐salt animals, along with histomorphometric evaluation that demonstrated tubular dilation, reduction of tubular epithelial cell height, an increase in interstitial cells, and a reduction in the cells' intratubular area. Together, these alterations may be related to a compensatory mechanism for tubular injury, especially of the proximal tubule. However, if the injurious stimulus persists, these same processes can transition toward maladaptive outcomes, including sustained loss of function, tubular changes, and progression to interstitial fibrosis, strengthening the relationship between structural alterations and their functional consequences.

Additionally, we investigated new mediators of renal damage that have been the subject of studies in recent years. LDH activity increases in the third week of DOCA‐salt treatment may be indicative of increased metabolic demand in response to water and salt overload. Altered urinary LDH has already been described as a marker of renal injury (Nielsen et al., 2017; Tsuji et al., 2025). Interestingly, urinary GGT activity was higher in both induction periods, suggesting tubular impairment. In fact, this marker is being postulated as an early mediator of tubular injury (Osborn & Fink, 2010; Peruchetti et al., 2021).

Other analytes measured in the present study are also being used as early markers of acute and/or chronic kidney injury (Brobak et al., 2024). NGAL levels were positively correlated with systolic blood pressure and negatively correlated with urinary sodium excretion (Brobak et al., 2024). Although we did not find a statistically significant difference when compared to control animals, this analyte was elevated in DOCA animals, suggesting impaired renal tubular function during the early phase of hypertension development.

Despite that, urinary levels of Cystatin C and β2M, low‐molecular‐weight proteins eliminated exclusively by glomerular filtration and catabolized by the proximal tubules, are increased in DOCA animals, suggesting impairment in tubular function (Zhang & Parikh, 2019). Consistent with these findings, the present study also demonstrated a reduction in the height of tubular epithelial cells of animals treated with DOCA. This, combined with the presence and elevated activity of GGT in the urine of DOCA animals, an enzyme present in the brush border of the renal epithelium, suggests impaired tubular function.

The kidney is a target organ for hypertension and may be a signal for the amplification of the sympathetic response observed in DOCA‐salt animals, which may lead to changes in glomerular and tubular functions. Classically, a urinary biomarker for kidney injury is albumin associated with a glomerular barrier lesion. Smaller proteins are filtered in the proximal tubule; impairment of this process may result in tubular proteinuria (Beckmann et al., 2025). In fact, the spatiotemporal organization along S1–S3 tubular segments with a multiple‐step protein degradation process may imply that tubular proteinuria may underestimate the degree of tubular lesion. Albumin levels did not differ among groups and periods evaluated despite the change in blood pressure levels. In addition, the histological analysis does not indicate glomerular impairment. Thus, our study supports the hypothesis that increased renal sympathetic activity is associated with preferential tubular dysfunction, given the absence of changes in creatinine and urea urinary levels. Moreover, GGT activity, an indicator of tubular damage, was altered in the periods evaluated. These renal tubular lesions may contribute to the severity of DOCA‐Sal induced hypertension. Both glomeruli and tubules have a complex innervation system, with different responses and signaling that have not yet been identified. Further studies are needed to address the role of afferent and efferent renal nerve fibers controlling the function of different segments of the nephron.

## 5 PERSPECTIVES

Overall, our findings show that modifications in renal mediators are related to the autonomic imbalance presented by DOCA‐salt animals. These alterations occur before a severe increase in blood pressure levels and are mostly of tubular origin, indicating potential urinary biomarkers that could be used in clinical practice to identify patients who would benefit from renal denervation for the control of refractory hypertension.

## AUTHOR CONTRIBUTIONS

**Edina Da Luz Abreu:** Conceptualization; data curation; formal analysis; investigation; methodology; validation. **Rafael S. Carvalhal:** Investigation; methodology. **Ana Caroline Marreiros:** Methodology; visualization. **Stephanie F. Gerolin:** Methodology. **Laura Barroso F. Oliveira:** Methodology; visualization. **Maria Eduarda Abreu‐Cunha:** Methodology; visualization. **Diogo B. Peruchetti:** Investigation; methodology; validation; visualization. **Cristiane D. Gil:** Methodology; validation. **Cássia T. Bergamaschi:** Conceptualization; data curation; formal analysis; methodology; project administration; validation. **Ruy R. Campos:** Conceptualization; data curation; formal analysis; funding acquisition; project administration; supervision; validation; visualization.

## CONFLICT OF INTEREST STATEMENT

No conflicts of interest, financial or otherwise, are declared by the author(s).

## ETHICS STATEMENT

All experiments were approved by the Ethics Committee on Animal Use (CEUA) of the Universidade Federal de São Paulo (CEUA/Unifesp, process no. 3812260824). All experimental approaches applied in this study followed the guidelines recommended by the National Council for the Control of Animal Experimentation (CONCEA, Brazil).

## Data Availability

Data sets were deposited in the Institutional Repository of Universidade Federal de São Paulo (UNIFESP), available at the following link: https://hdl.handle.net/20.500.12682/rdp/3HADDC.
